# Influence of surgeon experience on the incidence of tip fold-over with slim preformed cochlear implant electrodes

**DOI:** 10.1007/s00405-025-09235-w

**Published:** 2025-02-01

**Authors:** Esther Knörle, Caterina Vazzana, Timo Stöver, Silke Helbig

**Affiliations:** Department of Otorhinolaryngology, Head and Neck Surgery, Goethe University Frankfurt, University Hospital, Theodor-Stern-Kai 7, D-60590 Frankfurt am Main, Germany

**Keywords:** Cochlear implantation, Tip fold-over, Surgical experience, Preformed electrode - perimodiolar, Slim modiolar

## Abstract

**Purpose:**

To assess whether increasing experience with implantation of a thin preformed electrode array for perimodiolar insertion reduces the incidence of tip fold-over (TFO).

**Methods:**

The retrospective study included 100 patients who received a cochlear implant (CI) with the Slim Modiolar (SM) electrode array (Cochlear, Sydney, Australia) at a university CI centre between November 2015 and December 2022. Postoperative radiological imaging was performed to verify electrode position. Surgical reports and radiological images were reviewed and the incidence of TFO was analyzed for three experienced CI surgeons. In addition, the incidence of intraoperative measurements showing evidence of electrode malposition and the mean duration of surgery over time were documented.

**Results:**

129 SM implantations in 100 patients were included. In seven cases (5.4%) TFO was radiologically detected and successfully revised. In eight cases (6.2%), electrophysiological measurements indicated misplacement and the position was corrected during the same surgery. For one surgeon, five out of 67 implantations (7.5%) were affected by TFO, with the frequency of this complication decreasing over time. The average surgery time for all surgeons was 122.2 (± 44.2) minutes, with two surgeons showing a decrease over time.

**Conclusion:**

The results show a tendency that the SM electrodes can be implanted with a lower complication rate and faster over time. Therefore, it can be assumed that the implantation of the SM electrode requires a certain amount of practice, even for experienced surgeons. As intraoperative electrophysiological measurements detected 71.4% of all radiologically confirmed TFOs, their use is highly recommended.

## Introduction

Cochlear implants (CI) are electronic prostheses designed for the inner ear to restore speech perception in patients with sensorineural hearing loss who cannot be adequately managed with conventional hearing aids [1]. The accurate positioning of the electrode array within the cochlea is a critical factor in achieving optimal speech understanding [2, 3]. Currently, there is a wide variety of implants and electrode carriers available. These can be categorized into two different designs: straight and preformed electrode arrays. Straight electrode arrays are designed to slide along the lateral wall of the cochlea during insertion, whereas preformed electrode arrays are intended to achieve a position close to the modiolus. The insertion of preformed electrode arrays is more delicate due to their curvature and therefore requires an insertion device, a stylet or, as in the case of the *Slim Modiolar* (SM; Cochlear, Sydney, Australia), an insertion sheath [4].

Both electrode array designs are intended for insertion into the scala tympani. This recommendation is supported by studies demonstrating improved speech intelligibility [5, 6] and a lower incidence of vertigo with insertion into the scala tympani compared to the scala vestibuli [4]. Achieving this outcome is more reliable when using a round window approach [6].

The main reason for using preformed electrodes is their ability to better target stimulation of auditory nerve fibers, leading to improved frequency discrimination [7, 8]. Heutink et al. reported higher speech perception outcomes with precurved electrode implantation [3]. Over the past decade, straight electrodes have been regarded as more suitable for the preservation of residual hearing, particularly in the low frequency range [8]. In a study by Suhling et al. evaluating 511 implantations, hearing preservation was significantly lower with the previously available preformed electrodes (21%) compared to lateral wall electrodes (49%). This may be due to lateral wall electrodes being more likely to respect the boundaries of the scala tympani, minimizing trauma to inner ear structures [3, 9]. Preformed electrode carriers have been described in the literature as being more prone to causing inner ear trauma [4, 10]. For example, kinking of the electrode tip was reported more frequently with preformed electrode arrays inserted via a retractable sheath than with straight electrodes [11, 12]. However, most of these findings refer to earlier generations of electrode arrays and newer, slimmer arrays may offer a different perspective on hearing preservation. In terms of patient counselling, the key question arises as to whether increasing experience with the implantation of this new type of preformed slim lead reduces the incidence of the complications during surgery. Understanding this could significantly influence electrode selection and raise considerations regarding the surgeon’s expertise.

Therefore, the aim of this study was to assess, for the first time, whether the incidence of tip fold-over (TFO) decreases over time as surgeons become more experienced with the implantation of SM electrodes, which were launched in 2015.

## Materials and methods

### Patients

A total of 100 patients (47 women, 53 men) who underwent cochlea implantation at the otolaryngology department of a university hospital between November 2015 and December 2022 were included. All 100 patients received the SM electrode (either *CI 532* or *CI 632*) with insertion performed using a sheath according to the manufacturer’s guidelines. 71 patients received a unilateral CI, while 29 patients received bilateral CI. In total, 129 ears were included in the analysis. This retrospective study was approved by the local ethics committee.

### Surgeons

By November 2015, all three surgeons had accumulated over 10 years of experience in cochlear implantation. Each of them had performed more than 800 implantations during their careers.

### Radiological imaging

All patients underwent imaging on the first postoperative day to verify the correct positioning of the electrode array. For adults, digital volume tomography (DVT) using the *ProMax-3D SCS* system from PLANMECA was preferred. In cases where children were unable to remain still for DVT, Stenvers imaging with the *Philips Optimus Carestream DRX-1* was used. If detailed postoperative imaging with fewer motion artefacts was required, computed tomography (CT) scan of the petrous bone was performed using the *Somatom Definition AS +* or *Somatom Force* system from Siemens. Special attention was given to the detection of TFO during image evaluation.

### Statistical analysis

The number of intraoperative TFO events was assessed for each surgeon (named A, B, C). The frequency of this complication over time was analyzed for surgeon A to investigate a possible learning effect with SM electrode implantation. Therefore, the number of TFOs was recorded in chronological order based on the sequence of implantations performed.

To perform the statistical analyses in this study, the open-source software JASP (Jeffrey’s Amazing Statistics Program) was utilized. JASP offers a broad range of statistical methods, including t-tests, descriptive analyses, and regression analyses, providing a modern alternative to traditional tools like SPSS for teaching and research.

Logistic regression was applied to analyze a potential learning effect of surgeon A concerning the occurrence of tip fold-over (TFO) events over time. This statistical method examines the relationship between one or more independent variables (predictors) and a categorical dependent variable (target variable). It estimates the probability of a specific event occurring. In this study, time served as the predictor, and the occurrence of tip fold-over was the target variable [13].

The logistic regression model calculated a coefficient for the dependent variable, which indicates whether the probability of the event changes over time. A time coefficient less than zero suggests a decreasing probability over time, greater than zero suggests an increasing probability and equal to zero indicates no temporal influence on the event’s probability [13].

Additionally, the odds ratio (OR) was calculated to quantify the influence of independent variables (e.g., time) on the likelihood of the event occurring. An OR less than 1 indicates that the event is less likely to occur under the influence of the independent variable. The 95% confidence interval of the OR was also reported, providing a range where the true OR is expected to lie with 95% confidence. A narrow interval suggests a precise estimate, while a wide interval indicates uncertainty. If the confidence interval includes 1, the effect is considered not statistically significant. For example, in the analysis of tip fold-over events, a lower limit of 0.883 and an upper limit of 1.004 for the interval would indicate a non-significant effect due to the interval containing 1 [13, 14].

In addition, surgical reports were reviewed for cases where TFO was thought to be present intraoperatively and corrected during the same procedure.

Data on the duration of the implantation procedure were systematically collected. The surgery time was recorded in minutes, measured from the moment of incision to the completion of suturing. This information was documented with minute-level precision by the surgical nurse using a standardized digital documentation system employed in the clinic.

Linear regression was used to analyze the relationship between surgery time and years of experience, represented by the chronological number of implantations performed for each surgeon. Surgery time (in minutes) was the dependent variable, while the number of implantations performed served as the independent variable. The regression coefficient describes the direction and magnitude of the influence of the independent variable on the dependent variable. A regression coefficient less than zero indicates a negative correlation, where surgery time decreases as experience (number of implantations) increases. The coefficient’s value reflects the average change in surgery time for each unit increase in the independent variable. The significance of the relationship was assessed using the *p*-value, which tests whether the observed correlation is statistically significant [13, 14].

## Results

### Demographic data

The retrospective analysis included 100 CI users with 129 ears who received an SM electrode array between November 2015 and December 2022. The study population comprised 47 females and 53 males, with 61 (47.3%) implantations performed in female and 68 (52.7%) in male patients. The mean age at implantation was 50.9 (± 23.8) years, ranging from 0.6 to 86 years. The mean age was 49.7 (± 26.2) years for men and 52.3 (± 21) years for women.

### Incidence of tip fold-over

Electrode positioning was assessed by DVT in 99 cases (76.7%), X-ray in 23 cases (17.8%) and CT of the temporal bone in 7 cases (5.4%). In seven of the 129 cases (5.4%), a TFO was radiologically confirmed. All cases underwent successful revision surgery, with a different electrode (*Contour;* Cochlear, Sydney, Australia) being used in six cases. Intraoperative measurements suggested 13 cases of electrode TFO (10.1%). In eight cases (6.2%), the surgeons were able to correct the presumed electrode misplacement during the same procedure. In five cases, radiological confirmation of TFO was obtained postoperatively. This means that two of the seven radiologically confirmed TFOs were not detected at implantation. The last radiologically confirmed TFO in the study population occurred on 12 December 2017, approximately two years after the first procedure in November 2015.

To evaluate the complication rate per surgeon over time, only radiologically confirmed TFOs were included. Of the three surgeons, surgeon A performed 67 implantations, surgeon B performed 34 and surgeon C performed 28. The incidence of radiologically confirmed electrode TFO was 7.5% (5/67 implantations) for surgeon A, 2.9% (1/34 implantations) for surgeon B, and 3.8% (1/28 implantations) for surgeon C. For surgeon B, the TFO occurred during the 14th out of 34 implantations, and for surgeon C, it occurred during the 13th of 28 SM surgeries. As only one radiologically confirmed TFO occurred in surgeons B and C surgeries, no conclusions can be drawn about the incidence of this complication over time. For surgeon B, the complication occurred in surgery number 14 out of 34 procedures, and for surgeon C it occurred in surgery number 13 out of 28 procedures.

**Fig. 1:**
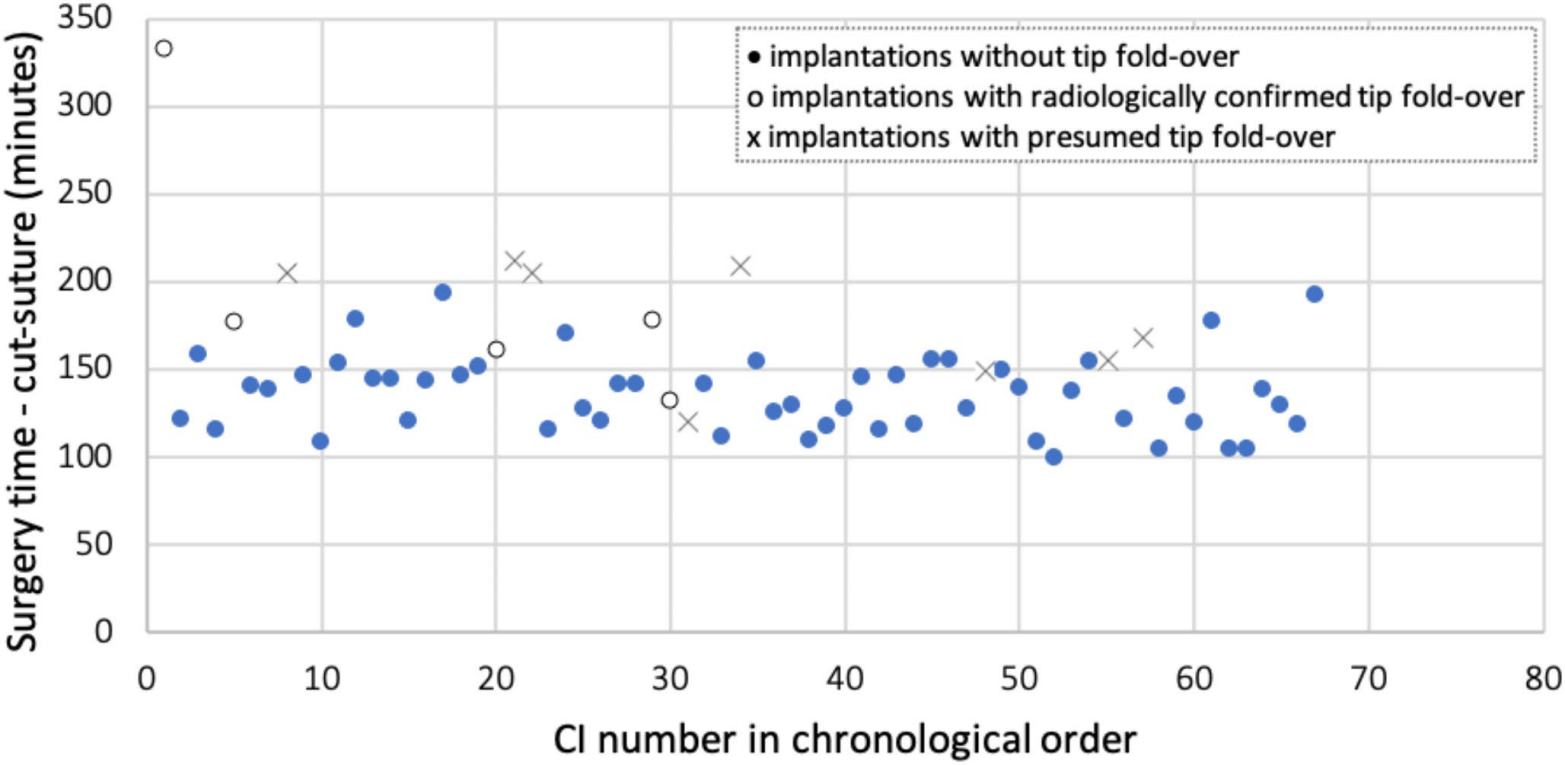
Surgeon A: Incidence of radiologically confirmed tip fold-over (circles) and presumed tip fold-over (crosses) over time, presented as the number of surgeries in chronological order. A tendency towards a reduction in both confirmed and presumed tip fold-overs was observed, although this did not reach statistical significance. Implantations without tip fold-over are shown as filled dots

Surgeon A had the last radiological evidence of a TFO at surgery number 30 out of a total of 67 procedures. Within the series, the last suspected TFO occurred at surgery number 57. For surgeon A, 7.5% (5/67) of the implantations resulted in a confirmed electrode TFO. A logistic regression analysis was performed with the incidence of this complication as the dependent variable and the number of implantations performed in chronological order as the predictor (Fig. 1). A negative association was observed between the occurrence of a TFO and the number of consecutive implantations (regression coefficient = -0.06, OR = 0.941, 95% confidence interval = 0.883, 1.004). Although this did not reach statistical significance (*p* = 0.066), there was a tendency for the risk of this complication to decrease with increasing surgical experience. An odds ratio of 0.941, which is less than 1, suggests that the likelihood of TFO decreases over time, indicating a reduction in risk with increasing experience.

In addition, the results for surgeon A were evaluated by including both presumed and confirmed TFOs. A total of eight presumed TFOs were identified and another five were confirmed, resulting in 13 cases. A logistic regression analysis with the incidence of a complication as the dependent variable and the number of implantations performed as the independent variable (Fig. 1) showed a negative correlation (regression coefficient = -0.021). Again, the risk of such complications decreased with experience, although this association did not reach statistical significance (*p* = 0.207), and the overall risk was lower when both presumed and confirmed TFOs were included in the analysis.

### Duration of surgery

The mean total surgery time of the 107 unilateral cochlear implantations was 122.2 (± 44.2) minutes, while the mean surgery time for the 11 bilateral cochlear implantations (22 ears) was 248.1 (± 46.7) minutes. The seven revision surgeries due to TFO required an average surgery time of 57.9 (± 23.3) minutes.

The mean duration of CI surgery varied among the three surgeons. For a total of 57 unilateral implantations, surgeon A had a mean surgery time of 146.1 (± 38.2) minutes, while surgeon B’s mean time was 101,4 (±39.6) minutes for 24 surgeries, and surgeon C’s mean time was 88.8 (± 26.3) minutes for 26 surgeries. For bilateral implantations, the mean duration of surgery was 277.2 (± 40.1) minutes for surgeon A (10 ears) and 232.2 (± 30.6) minutes for surgeon B (10 ears). Surgeon C performed only one bilateral implantation which took 173 min.

**Fig. 2:**
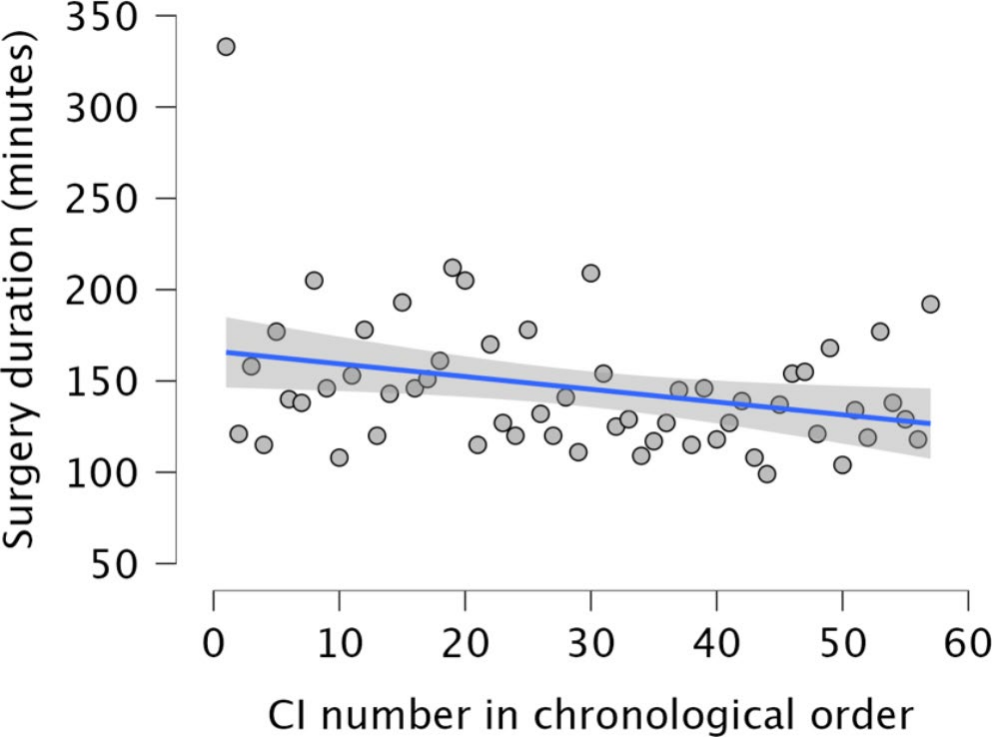
Surgeon A: Linear regression analysis of cochlear implantation duration over time yielded a coefficient of -0.37. The first, unusually prolonged intervention with the new electrode is shown but was excluded from the analysis. Although the *p*-value of 0.124 was not statistically significant, this suggests a tendency for the average duration of surgery to decrease with each implantation

The difference in average surgery time per surgeon over time was analyzed using linear regression, with surgery time in minutes as the dependent variable and the number of SM electrode implantations in chronological order as the independent factor. For surgeon A, the first implantation, lasting 333 min, stood out from the subsequent implantations, which ranged from 99 to 212 min. According to the surgical records, this was probably due to the presence of the manufacturer during the initial procedure and prolonged hemostasis. The linear regression model was found to be reasonably applicable when the first procedure was excluded. The regression coefficient was negative at -0.37, indicating a trend towards shorter surgery duration with successive procedures, although this was not statistically significant (*p* = 0.124). It was estimated that the duration of implantation decreased by an average of 3.7 min after every 10 procedures. Figure 2 includes the first surgery with a duration of 333 min to illustrate the marked difference between this case and subsequent surgeries.

**Fig. 3:**
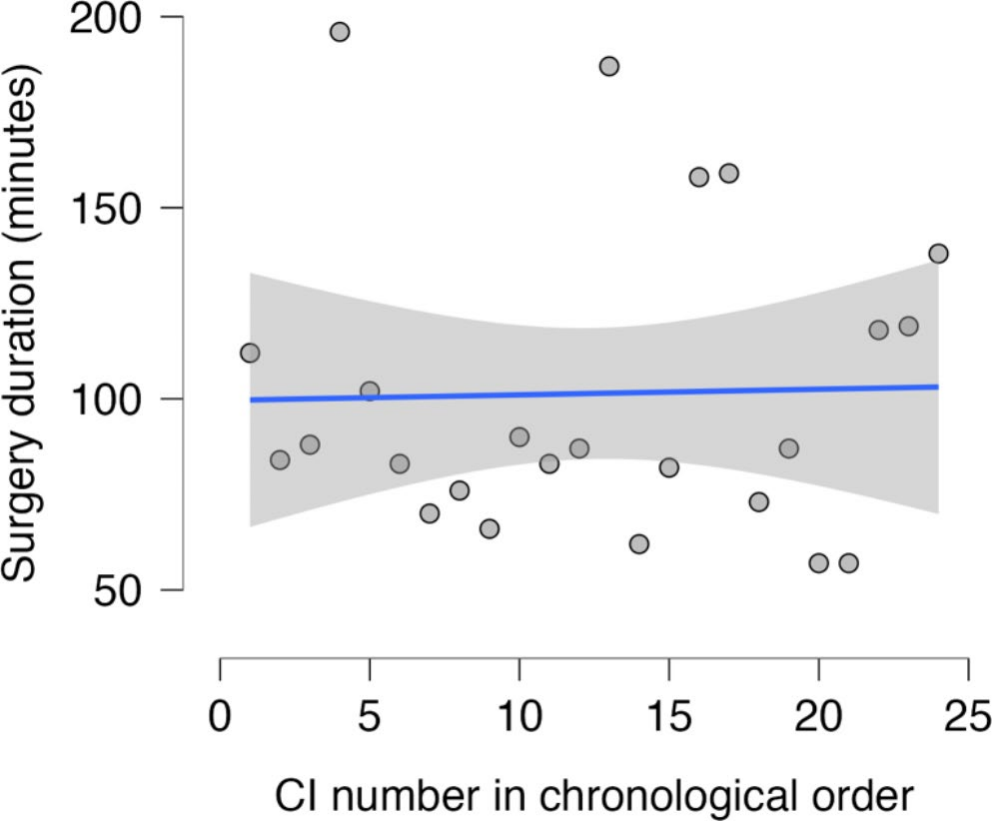
Surgeon B: Linear regression of surgery time (in minutes) over time showed a positive regression coefficient of 0.147, indicating a statistically non-significant increase in average surgery time (*p* = 0.903)

For surgeon B, the regression coefficient was positive at 0.147, with a non-significant *p*-value of 0.903, indicating no reduction in surgery time over time (Fig. 3).

**Fig. 4:**
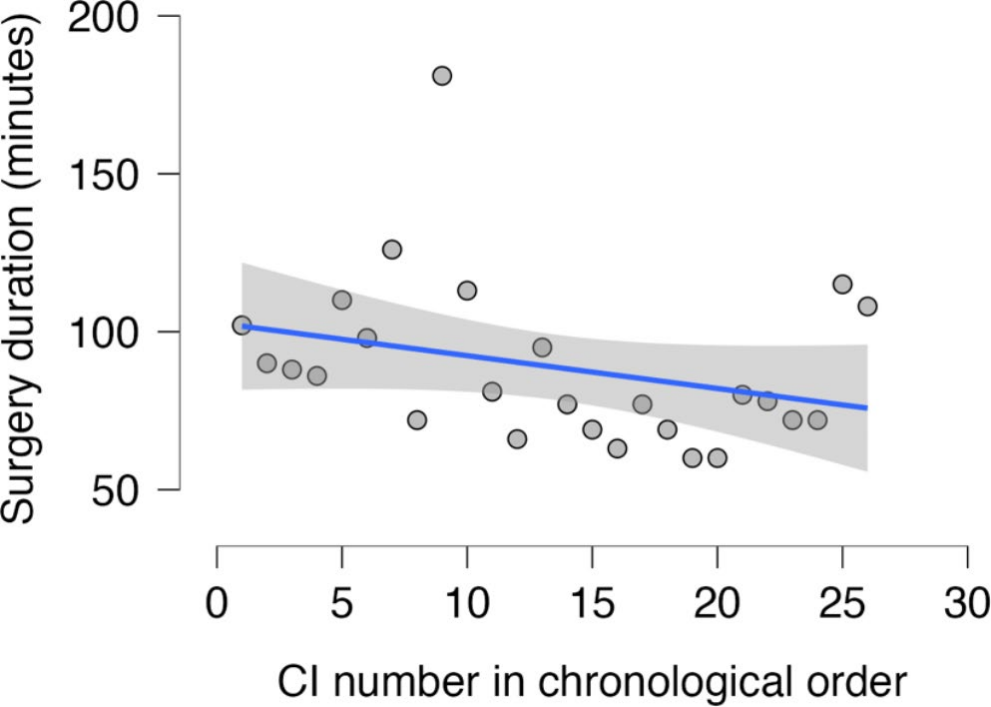
Surgeon C: Linear regression of surgery time (in minutes) over time showed a negative regression coefficient of -1.039. Although not statistically significant (*p* = 0.133), this indicates that the average time taken to perform consecutive surgeries tends to decrease

For surgeon C, the regression coefficient was negative at -1.039, and although not statistically significant (*p* = 0.133), it suggested a decrease in average surgery time over time (Fig. 4). Based on the linear regression model, it is estimated that after 10 implantations, surgery time decreased by an average of 10.4 min.

## Discussion

The results presented here deal with surgeons’ first steps in implanting an improved electrode carrier design that has been available since 2015. The complication of TFO was observed in 5.4% of cases. All surgeons had extensive experience (> 15 years) in cochlear implantation, especially with preformed electrodes from the same and other manufacturers, and all surgeons had undergone the training provided by the manufacturer. Therefore, it can be assumed that the first use of an improved electrode array was the only factor influencing the results in this study. Logistic regression analysis of TFO incidence over time showed that the risk of radiologically confirmed TFO decreased with increasing surgeon experience.

To the best of our knowledge, there are no comparable studies in the literature evaluating the incidence of TFO in experienced surgeons over time as they gain initial experience with implanting an improved electrode design of a thin preformed electrode array for perimodiolar insertion.

However, it has been suggested that electrodes designed for perimodiolar placement may be more prone to this complication [12]. A review by Dhanasingh et al. summarised the incidence of TFO described in a total of 13 studies up to 2018, covering all electrode types. In a total of 3177 implantations, the TFO rate was 1.6%, affecting 50 ears [15]. Looking at the SM electrode specifically, the incidence of TFO reported in the literature ranges from 4.1 to 6% [16–19], suggesting an increased susceptibility to complications with this thin electrode design. Our data are therefore consistent with the literature, which shows a comparable incidence of 5.43%, based on 129 implants performed.

It is therefore important to counsel CI candidates about this specific risk. Surgeons with more experience in implanting the SM should be consulted to further reduce this complication. It may even be worth considering restricting the implantation of SM electrode arrays to experienced surgeons in larger centres performing a higher number of implantations, as a higher number of SM cochlear implantations will have an impact on the level of complications. The results presented here showed that approximately 30 implantations would be required before there were no more radiologically detectable TFOs. The findings of this study suggest that less experienced surgeons may need special training to successfully implant preformed electrode arrays and prevent complications. Supervision by more experienced surgeons may be important and helpful at this stage.

While there are studies on the average duration of surgery for cochlear implantation, no studies have examined the duration of surgery for the SM electrode over time. In a study by Cuda et al., the average time for a total of 67 implantations using the *CI 532* SM electrode was investigated. The average surgery time per surgeon was 58.7 (± 8.3) minutes. With a range of 50 to 75 min, these times were shorter than those of the surgeons in this study, who had an average of 123.3 (± 44.9) minutes for 109 unilateral implantations [20]. This discrepancy could be explained by additional technical and physiological measurements during the surgery, such as a technical check of the CI immediately prior to implantation, or by slight differences in the surgical procedure. The fact that we found differences in the average duration of surgery between surgeons A, B and C, as well as the results in the literature, suggests that valuable measurements can only be made by the analysis of individual changes over time.

There was a tendency for the duration of surgery to decrease over time in this study. All surgeons were experienced in implanting preformed electrode arrays and the procedure prior to insertion of the enhanced electrode design did not differ from previously performed cochlear implantations. Therefore, it can be assumed that new handling skills need to be developed for the modified insertion procedure using a sheath and a preformed electrode carrier, which should be implanted in the correct orientation towards the modiolus as suggested by the manufacturer of the SM electrode. These skills will improve with experience and become more efficient. Furthermore, intraoperative measurements and patient-related complications such as prolonged bleeding may influence the duration of the procedure. Future studies specifically documenting insertion time alone would be necessary to determine whether surgeons reduce their insertion time when implanting the SM electrode for the first time.

In a total of 13 implantations, intraoperative “transimpedance matrix” and “spread of excitation” measurements indicated TFO. Radiological confirmation of this complication was obtained in five of these cases. This is in agreement with the findings of Grolman et al. who detected four cases of TFO in 72 implanted ears intraoperatively using DVT due to irregular intraoperative measurements [21]. These electrophysiological tests appear to be valuable tools for the assessment of TFO immediately after electrode placement [22–24]. Coping strategies such as these contribute to the achievement of correct electrode placement during cochlear implantation by helping to identify problems within the intervention, thereby reducing the need for revision surgery.

Although this study only highlights the complication of TFO seen with implantation of a preformed, very thin lead over time, it must be considered that other lead designs also have the potential for complications. For example, lateral wall electrodes have been associated with electrode migration in some cases [25]. Clearly, different designs require different management strategies and most likely different numbers of procedures to achieve optimal results.

The need to identify problems with newly introduced electrode carriers and possible insertion complications in general therefore argues for quality control in cochlear implant centres. The additional use of registries will most likely help to identify complications that are limited to the specific electrode designs that are used, as well as individual problems of surgeons or centres [26, 27]. Ultimately, the safest treatment for patients will be achieved through transparent communication.

## Limitations

In this study, only one surgeon had a sufficient number of SM implantations to statistically evaluate complications. Therefore, there is a need for a higher number of procedures per surgeon or even better multi-centre studies to assess whether the reduction in complications such as TFO is a more widespread issue rather than specific to a single surgeon or centre. In particular, the average number of implantations required to eliminate or at least significantly reduce the complication of TFO would be important. In terms of the statistical power of the results, a larger number of implantations would increase the statistical power and contribute to more robust conclusions.

Since a second surgical nurse was assigned to assist outside the sterile area during each cochlear implantation, particularly to document the standardized surgical protocol, the recorded surgery time can be considered accurate. However, the inclusion of electrophysiological measurements of the CI, which can vary in duration, may influence the overall surgery time. These variations in measurement cannot be entirely ruled out and should be considered when interpreting the recorded duration of the procedure.

## Conclusion

This study of 129 implantations with the SM electrode carrier shows a tendency for the incidence of TFO to decrease over time. Assuming that experience affects SM implantation, it may be worth considering whether implantation of this thin, perimodiolar electrode should be restricted to experienced centres with a higher number of implantations, or at least supervised by an experienced surgeon.

In addition, although not statistically significant, the mean duration of surgery was found to decrease with increasing experience in this study. In conclusion, besides the general training provided by the manufacturer, it is reasonable to recommend specific ongoing training according to the manufacturer’s guidelines when using the SM for the first time, and probably also when using other preformed electrode carriers with different insertion mechanisms.

Future studies should focus on the identification of common complications based on electrode type and should attempt to determine the number of implantations required to achieve the safest level of cochlear implantation. In order to reduce the risk of complications such as in this study TFO with the SM electrode, appropriate training of surgeons and a sufficient number of implantations with a specific electrode type are advisable.
